# Response of the Cardiac Autonomic Control to Exposure to Nanoparticles and Noise: A Cross-Sectional Study of Airport Ground Staff

**DOI:** 10.3390/ijerph18052507

**Published:** 2021-03-03

**Authors:** Luigi Isaia Lecca, Gabriele Marcias, Michele Uras, Federico Meloni, Nicola Mucci, Francesca Larese Filon, Giorgio Massacci, Giorgio Buonanno, Pierluigi Cocco, Marcello Campagna

**Affiliations:** 1 Department of Medical Sciences and Public Health, University of Cagliari, 09042 Monserrato, Italy; gabriele.marcias@libero.it (G.M.); michele_uras@hotmail.com (M.U.); federicomeloni@hotmail.it (F.M.); pcocco@unica.it (P.C.); mam.campagna@gmail.com (M.C.); 2 Department of Experimental and Clinical Medicine, University of Florence, Largo Brambilla 3, 50134 Florence, Italy; nicola.mucci@unifi.it; 3 Department of Civil and Environmental Engineering and Architecture, University of Cagliari, via Marengo 2, 09123 Cagliari, Italy; massacci@unica.it; 4 Unit of Occupational Medicine, Department of Medical Sciences, University of Trieste, 34129 Trieste, Italy; larese@units.it; 5 Department of Civil and Mechanical Engineering, University of Cassino and Southern Lazio, via Di Biasio 43, 03043 Cassino, Italy; buonanno@unicas.it

**Keywords:** UFP, nanoparticles, noise, HRV, autonomic control, short-term health effect

## Abstract

Airport activity causes the emission of particulate matter and noise, two environmental contaminants and potential health hazards, particularly for the personnel operating nearby taxiways. We explored the association between exposure to fine/ultrafine particles (UFPs) and noise with heart rate variability (HRV), an early indicator of cardiovascular autonomic response, among a sample of airport ground staff. Between May and June 2018, thirty-four male operators (mean age = 43 years and SD = 6.7) underwent personal monitoring of exposure to nanoparticles and noise, and HRV during their work activity. We conducted univariate and multivariate analysis to test the effect of UFP and noise exposure HRV. Total Lung Deposition Surface Area (LDSA) was significantly associated with a decrease in HRV Total Power and Triangular index (*β* = −0.038 *p* = 0.016 and *β* = −7.8 × 10^−5^, *p* = 0.042, respectively). Noise peak level showed an opposite effect, which was significant for Total Power (*β* = 153.03, *p* = 0.027), and for Triangular index (*β* = 0.362, *p* = 0.035). Further investigation is warranted to clarify the effect of the concurrent exposure to UFPs and noise on early changes of cardiac autonomic regulation.

## 1. Introduction

Several studies have suggested airports as a potential source of emission of particulate matter and noise in the surrounding environment [1,2]. Therefore, airport ground staff, such as firefighters, flight security agents, and aviation fuel’s administration staff (AFS), might be more severely exposed [1,3].

Particulate matter has been associated with cardiovascular and respiratory effects [4,5], and with a potential carcinogenic effect for the lung [6]. Nevertheless, it is not clear yet whether such effects depend upon the specific chemical properties of the particulate matter, or on the physical properties, such as size, morphology, surface area, and charge, of inhalable particles [7,8,9].

Several studies have evaluated the autonomic balance of the cardiovascular system, in relation to exposure to fine/ultrafine particulate, in both animal [10] and human models [11,12]. Those studies confirmed a reduction in heart rate variability (HRV).

Long-term exposure to environmental noise is known to affect the cardiovascular system, by contributing to the development of hypertension, ischemic heart disease, and stroke [13,14]. Acute exposure, on the other hand, elicits the autonomic nervous system and endocrine system response, with release of catecholamine and glucocorticoid, resulting in increasing systolic and diastolic blood pressure, and heart rate [13,15].

Heart rate variability (HRV) is a valid method for measuring the response of the autonomic heart control to noise, through the changes induced in some HRV parameters [16,17].

Environmental noise is a well-known risk factor for the airport ground staff and for the general population in the airport surroundings [3]. Several studies reported an association between exposure to noise from air traffic and cardiovascular effects, such as an increased risk of myocardial infarction and stroke, and of hospitalization for cardiovascular diseases among subjects living in the surroundings of civil airports [18,19].

Studies on the combined effects of noise and air pollution showed largely independent effects, in relation to the different mechanisms by which either exposure can cause detrimental effects on human health [20,21,22]. Since both ultrafine particles and noise might target the cardiovascular system, detecting the early effects on the cardiac autonomic regulation of the combined exposure would help to clarify whether an additive or synergic mechanism occurs.

The present study aimed to investigate whether environmental exposure to fine/ultrafine particulate and noise might affect HRV, as an early indicator of cardiovascular effects. To match the objective, we did the following: (i) performed personal monitoring of exposure to ultrafine particles (UFPs) during flight attendance; (ii) assessed personal exposure to impulsive and continuous noise; (iii) monitored heart rate variability (HRV) during the working shift; and (iv) evaluated the impact of UFPs and noise exposure on HRV changes.

To the best of our knowledge, the study herein presented is the first exploring potential early effects on the cardiovascular system, due to the combined exposure to fine/ultrafine particles and noise in a sample of airport ground staff.

## 2. Materials and Methods

### 2.1. Study Design

Between March 16th and May 18th 2018, we conducted a single, simultaneous personal monitoring of noise and airborne UFPs and HRV lasting no less than two hours, between 7:30 a.m. and 8:00 p.m. during a regular working day in the local airport. Every operational day, we monitored two or three study subjects.

With the aim of reducing, if possible, any interference with the operational activities, the monitoring program was planned with one-week intervals, and after consulting the supervisor for the internal organization and the scheduled airport activities. 

The study was conducted as an observational, cross-sectional study on subjects recruited among the ground operating airport personnel, through an in-site recruitment accorded with supervisors. Study subjects had to be engaged in the following tasks: Aircraft Ground Staff (AGS), firefighting officer, flight security agent, and aviation fuel’s attendants (AFA). Participation was on a volunteer basis, following the signature of an informed consent form about the purpose of the study, prior to interview and the tests according to the study protocol. The study was conducted in accordance with the Helsinki Declaration. Subjects suffering from any cardiovascular, endocrine, or neurologic disease were ineligible for study. Overall, 34 male subjects (mean age = 43; SD = 6.7) were enrolled.

All participants had an electrocardiogram (ECG) at rest and their anthropometric and lifestyle (date of birth, height and weight, smoking habit, health status, and medicaments intake) recorded by a trained medical doctor, before undergoing the study protocol, during the regular working hours. Participants had to wear portable devices to monitor personal exposure to UFPs and noise, and a Holter ECG device while working. After setup, participants had to rest in the room for at least 5 min, to register background HRV parameters before exposure. Each subject received also a diary, for a step-by-step registration of the activities performed during the monitoring hours, with the corresponding time of starting and ending each.

The monitoring phase covered no less than two hours of a regular working day, after which participants had to return to the room and to rest for no less than 10 min, to register HRV at the end of the test. Data from the Holter ECG were saved on a dedicated laptop PC that was set up with the specific acquisition software. Afterwards, memory of the device was deleted, to allow for the recording of a new ECG.

### 2.2. Personal Exposure Assessment

The method for measuring noise and personal exposure to UFP was described elsewhere [3]. Briefly, noise was recorded using a BSWA Mod. MP201 (BSWA Tech., Beijing, China) microphone, connected to a Larson Davis PRM 828 (Depew, NY, USA) preamplifier. Microphone inputs were sent to a Larson Davis Model 820 (Depew, NY, USA) integrating sound level meter (1 Hz sampling rate). The microphone was placed on the helmet worn by each operator while working. Before and after each monitoring session, we calibrated the instruments, using a Larson Davis LD CAL 200 (Depew, NY, USA) device, to ensure measurement’s accuracy. The data recorded were exported to the NWWin (Noise & Vibration Works, Vimercate MB, Italy) software, for analysis. The following parameters were calculated for each participant: A-weighted noise exposure level (LAeq), A-weighted noise exposure level normalized to an 8 h working day (LAeq8hr), and Peak Sound C-weighted Pressure Level (LC peak). 

UFP was collected with a Diffusion Size Classifier instrument (DISCmini, Matter Aerosol, Wohlen, Switzerland). DISCmini is based on the electrical charging of the aerosols to determine the alveolar Lung Deposited Surface Area (LDSA, µm^2^/cm^3^), the average particle size (nm) in the 10–300 nm range, and the particle count (particles/cm^3^). 

Total LDSA is a parameter of cumulative exposure, and it is calculated with the following formula:

Total LDSA = mean LDSA × V(1)

LDSA: Lung Deposition Surface Area as µm^2^/cm^3^.

V: Sampled volume in liters.

The ECG tracks were recorded for the entire monitoring period using a three channel (five-lead) digital Cardiette Holter System equipped with the ECG Pilot and giOtto v.7.0.1.26 software.

Raw data on the normal RR interval (NN) were extracted from the archive, by the software giOtto; converted in a “.data” file; and then analyzed with the Kubios HRV standard v. 3.1.0 software, applying a medium-intensity filter to eliminate artifacts. The following HRV parameters were calculated:

- Standard Deviation of normal-to-normal (NN) intervals (SDNN, ms);
- Root Mean Square of successive differences in adjacent NN intervals (RMSSD, ms);
- Triangular index (T-index), corresponding to the integral of the density distribution divided by its maximum value;
- Very low frequency power (VLF, ms^2^) in the range 0.003–0.04 Hz;
- Low-frequency power (LF, ms^2^) in the range 0.04–0.15 Hz;
- High-frequency power (HF, ms^2^) in the range 0.15–0.40 Hz;
- LF/HF ratio (Ratio LF [ms^2^]/HF [ms^2^]);
- Total Power (TP, ms^2^): variance of NN intervals over the temporal segment, approximately in a range ≤ 0.4 Hz.

HRV parameters were determined over the first five minutes of registration (background activity), along the entire monitoring period, and for the last 5 min [23]. If more than 5% artifacts showed up in the last five minutes of the Holter ECG record, the previous five-minute segment was used to calculate the final HRV. Heartbeat annotations were automatically assigned by the software and reviewed by a trained physician. Only normal sinusal heartbeats were used for calculating the HRV parameters.

### 2.3. Statistical Analysis 

Descriptive statistics of the UFP and noise parameters were presented elsewhere [3]. The normal distribution was checked by the Kolmogorov–Smirnov test and visual inspection of the distribution curves. Log-transformed data were used for the following parameters: concentration, LDSA, total LDSA, VLF power, LF power, HF power, and Total Power.

The correlation between UFP parameters, noise exposure levels, and HRV parameters was calculated with the Pearson’s correlation test or the Spearman’s rho, as appropriate for parametric and non-parametric data, respectively. Background and final HRV parameters were compared with the *t*-test for paired data. Final HRV parameters were individually predicted by linear regression modeling, as a function of the UFP and noise-exposure parameters, adjusted for age, BMI, smoking habit, and hypertension, as the covariates. Finally, we tested the interaction effect of UFP and noise exposure by including the interaction term between total LDSA and Laeq8hr and Lc peak, respectively. Due to an instrument failure, the HRV was not correctly measured in one study subject, leaving the records of 33 subjects available for analysis.

The analyses were conducted by using SPSS (v. 20, package for Windows, SPSS Inc., Chicago, IL, USA). The null hypothesis was rejected when associated with an α-error of 0.05.

## 3. Results

Table 1 shows summary statistics of UFP and noise parameters of exposure in the study participants. The mean UFP concentration was 61 x 10^3^ particles/cm^3^ (SD = 351,475.20), while mean Total LDSA was 15.29 mm^2^ (SD = 23.08). Continuous noise levels did not exceed 80 dB (79.6 dB and SD of 7.4 for LAeq; 74.1 dB and SD of 7.8 for LAeq8hr), and mean peak noise levels was 129.3 dB (SD = 4.3).

Table 2 shows baseline and final HRV parameters. SDNN (*p =* 0.001), RMSSD (*p* < 0.001), and HF power (*p =* 0.049) were significantly decreased at the end of the sampling period, while VLF power showed a significant increase (*p =* 0.047).

There was a significant correlation between the HRV T-index and exposure to continuous (Pearson’s correlation coefficient *r* = 0.382; *p* = 0.031) and impulsive noise (*r* = 0.403; *p* = 0.022), but not with UFP exposure levels (Table 3). HRV Total Power was significantly correlated with impulsive noise (*r* = 0.366; *p* = 0.039) and age (*r* = 0.359; *p* = 0.040), but not with continuous noise level (*r* = 0.265; *p* = 0.143). HRV Triangular index was significantly correlated with age (*r* = 0.348; *p* = 0.047) and BMI (*r* = 0.388; *p* = 0.026), but not with continuous or impulsive noise nor UFP exposure.

UFP parameters showed a significant correlation with continuous noise parameters (*r* = 0.367, *p* = 0.039; *r* = 0.425, *p* = 0.015; *r* = 0.410, *p* = 0.022; for UFP concentration, LDSA, and Total LDSA, respectively), but not with impulsive noise parameters. Laeq8hr and Lc peak values were strongly correlated (*r* = 0.561; *p* = 0.001) (Table 3). 

The multivariate linear regression model predicting HRV parameters showed a significant effect of Total LDSA (*β* = −0.038, *p* = 0.016) and HRV T-index (*β* = −7.8 × 10^−5^, *p* = 0.042) on reducing HRV Total Power, and of Lc Peak on increasing both (Lc Peak: *β* = 153.03, *p* = 0.042 for Total Power; Lc Peak: *β* = 0.362, *p* = 0.035 for Triangular index). (Table 4). Age was also a significant predictor for a decrease in HRV Total Power (*β* = −92.95 *p* = 0.024), and marginally so for a decrease in T index (*β* = −0.187, *p* = 0.062). The multivariate analysis did not reveal any significant effect of UFP and noise on the SDNN, RMSSD, VLF, LF, HF, and LF/HF HRV parameters (data not shown).

Table 4 shows results of the best-fit models, including all noise and UFP parameters; using only one, namely total LDSA for UFP and Lc peak for noise, resulted in a substantial reduction in the R^2^ value, from R^2^ = 0.476 to 0.292 for HRV Total Power, and from R^2^ = 0.512 to 0.443 for HRV T index (not shown in the Tables). Including the interaction terms between LDSA and Lc peak did not reduce the residual variance of the regression model predicting HRV Total Power and Triangular index. Only the noise parameter and age confirmed their effect, suggesting that the effect of UFP exposure in this working population was confounded by concurrent exposure to noise.

## 4. Discussion

The purpose of our study was to test the association of the concurrent exposure to nanoparticles and noise and early changes in cardiac autonomic regulation, as detected by continuous monitoring of HRV parameters, during working activity in an airport.

Our findings suggest that impulsive noise may contribute to changes in the autonomic regulation of the heart immediately after exposure. We could not confirm the short-term effect of UFP on HRV previously reported in human studies [24,25,26], and in animal models [27].

The comparison between background and final HRV parameters showed a significant decrease for several time domain HRV indicators, such as SDNN and RMSSD, and a significant shift in frequency domain parameters, with an increase in VLF power and a decrease of HF power. Vagal activity is the major contributor to the HF component. A decrease in HF power might depend on a reduction in vagal activity [28].

UFP exposure in our study was higher than in similar studies (mean: 61,443 vs. 11,872 n/cm^3^), with a much larger Standard Deviation [3,24], as a result of a different exposure pattern, with short-term peak levels, followed by low background levels. Such an exposure pattern might account for our observation of lack of independent HRV effects. Besides, we tested a short-term effect by measuring HRV parameters immediately after UFP exposure, whilst other studies presented results of long-term HRV measurements, conducted several hours after UFP exposure [26].

Moreover, specific components of air pollution, besides particle size, might induce HRV changes. In our study, airport workers were exposed mainly to exhausts from aviation fuels and diesel vehicles, an exposure scenario quite different from road traffic [29] or welding [25].

Therefore, different UFP exposure patterns might explain the inconsistent reports, with some studies showing an association between UFP and changes in the HRV parameters [30,31,32], and other studies finding the opposite [33], while a clear interaction effect by UFP and noise, which we did not observe, is still uncertain.

In our study, impulsive noise, but not continuous noise, showed an association with the HRV Total Power and the Triangular index HRV parameters. This finding is consistent with previous studies [17] suggesting that different patterns of exposure to noise can differentially influence the cardiac autonomic control. Indeed, continuous noise exposure might increase some HRV parameters [34], also when considering co-exposure with UFP [35].

Our results suggest that the effect of exposure to UFP on short-term changes in HRV parameters among airport ground staff seems to be at least partially explained by concomitant exposure to impulsive noise.

Some limitations should be considered in interpreting our results. First, the peculiar exposure pattern, consisting in peak UFP levels on very low background levels, might not detect possible cumulative exposure effects. Second, our observation was limited to the exposure time and a short, immediately following, period; prolonging HRV monitoring for several hours after the end of exposure might have allowed us to observe any medium- and long-term effects on the autonomic cardiac regulation. Third, the study lacks consideration of physical activity during the test. However, during the test, study participants were mainly engaged in driving different vehicles (trucks, tractors, and shuttle buses), which requires a level of energy expenditure between 2.0 and 2.8 metabolic equivalents of task (METs), corresponding to a light-intensity activity [36]. Workers involved in moderate-intensity (3.0–5.9 METs) and vigorous-intensity (≥6 METs) physical activities, such as baggage handling (6.5–8.0 METs), were not included in the study population. Besides, the HRV evaluation considered only the last ten minutes of the ECG track, which was recorded while resting, isolated from external exposure, so to avoid the influence of physical load on HRV parameters. Nevertheless, further investigation with a physical activity assessment by means of an accelerometer could provide new insight on this relevant aspect. Finally, the study design lacks an unexposed reference group, to which HRV changes before and after the exposure period should be compared. Further investigations should compare subgroups of increasing exposure level to an unexposed reference group, so to confirm the putative role of UFP and noise exposure on decreasing or increasing HRV parameters.

## 5. Conclusions

In conclusion, our study of airport ground staff exposed to mean UFP concentration of 61,000 particles/cm^3^ and mean impulsive noise of 129 dB, conducted during a work shift, allowed us to highlight an association between exposure to impulsive noise and changes in HRV parameters, while concurrent exposure to noise would explain the observed association with UFP exposure. A decrease in HRV Total Power and Triangular index associated with impulsive noise exposure supports its influence on short-term cardiac autonomic control.

Further studies are warranted on monitoring personal exposure and early changes in cardiovascular control patterns, extended for several hours after exposure, to confirm the results of the present study.

## Figures and Tables

**Table 1:** Ultrafine particle (UFP) and noise exposure parameters in the overall sample of 33 participants.

UFP Parameters (N = 33)			
UFPs (part/cm^3^)	Size (nm)	LDSA (m^2^/cm^3^)	Dose LDSA (mm^2^)
Mean (SD)	Mean (SD)	Mean (SD)	Mean (SD)
61,443.30	55.77	109.46	15.29
(351,475.20)	(25.63)	(506.38)	(23.08)
**Noise Parameters (N = 33)**			
**LAeq (dB)**		**LAeq8hr (dB)**	**Lc peak (dB)**
**Mean (SD)**		**Mean (SD)**	**Mean (SD)**
79.606		74.112	129.773
(7.378)		(7.831)	(4.328)

LDSA, Lung Deposition Surface Area; LAeq, A-weighted noise exposure level; LAeq8hr, A-weighted noise exposure level normalized to an 8 h working day.

**Table 2:** Heart rate variability (HRV) parameters (mean, SD, and range) for basal and final time of sampling and *t*-test results for paired data between basal and final time sampling.

HRV Measures	Basal			Final			*t*-Test
	Mean	SD	Range	Mean	SD	Range	*p*
**SDNN**	58.85	17.72	90.60–26.20	48.15	17.09	19.70–86.30	**0.001**
**RMSSD**	59.22	59.22	18.50–107.5	43.22	19.36	13.90–92.20	**0.000**
**T-Index**	10.80	3.32	5.44–18.10	10.83	3.69	3.90–17.32	0.957
**VLF ms^2^**	165.1	129.4	18.00–465.0	234.49	230.0	14.00–852.0	**0.047**
**LF ms^2^**	1420.4	1103.7	344.0–4903.0	1409.88	1002.0	157.0–4660.0	0.955
**HF ms^2^**	846.0	809.9	40.00–3465.0	546.76	644.7	29.00–3193.0	**0.049**
**LF/HF**	3.46	4.58	0.37–24.61	4.43	3.30	0.45–14.35	0.265
**TP ms^2^**	2435.73	1703.77	540.0–6451.0	2194.58	1584.14	218.00–7010.00	0.405

SDNN, Standard Deviation of normal-to-normal; RMSSD, Root Mean Square of successive differences; VLF ms^2^, very low frequency power; LF ms^2^, low-frequency power; HF, ms^2^, high-frequency power; TP, Total Power.

**Table 3:** Correlation matrix between parameters of exposure to UFP and noise, HRV parameters, and individual characteristics.

	Age	BMI	UFP Concentration	UFP Size	LDSA	Total LDSA	LAeq8hr	Lc Peak	SDNN	RMSSD	T-Index	VLF Power	LF Power	HF Power	TP
Age	1	0.254	−0.040	−0.099	−0.027	0.014	0.014	0.048	−0.313	−0.173	−0.348 *	−0.347 *	−0.338	−0.298	−0.359 *
BMI		1	−0.225	−0.019	−0.252	−0.251	−0.493 **	−0.295	−0.288	−0.241	−0.388 *	−0.136	−0.174	−0.321	−0.230
UFP Concentration			1	−0.013	0.516 **	0.948 **	0.367 *	0.249	0.048	0.048	0.066	−0.004	0.011	0.049	0.002
UFP Size				1	0.043	−0.127	0.240	0.137	−0.037	−0.135	−0.006	−0.027	0.013	−0.159	0.000
LDSA					1	0.486 **	0.425 *	0.203	0.180	0.173	0.209	0.180	0.077	0.100	0.100
Total LDSA						1	0.410 *	0.396 *	0.112	0.117	0.110	0.011	−0.003	0.120	0.032
LAeq8hr							1	0.561 **	0.307	0.230	0.382 *	0.236	0.208	0.242	0.265
Lc Peak								1	0.325	0.293	0.403 *	0.332	0.323	0.308	0.366 *
SDNN									1	0.902 **	0.903 **	0.634 **	0.767 **	0.922 **	0.892 **
RMSSD										1	0.730 **	0.443 **	0.505 **	0.911 **	0.695 **
T-Index											1	0.664 **	0.836 **	0.817 **	0.903 **
VLF Power												1	0.723 **	0.529 **	0.759 **
LF Power													1	0.681 **	0.957 **
HF Power														1	0.843 **
TP															1

Note. * *p* < 0.05; ** *p* < 0.01.

**Table 4:** Multiple linear regression model to predict HRV Total Power and HRV Triangular index (T-index) as a function of UFP and noise exposure parameters, and of personal and lifestyle covariates.

	HRV Total Power		HRV T Index	
**Predictors**	*β* (se)	*p*	*β* (se)	*p*
**Constant**	−17795.8 (8627.2)	0.053	−34.652 (21.50)	0.122
**UFP Concentration**	0.229 (0.130)	0.092	0.003 (0.001)	0.418
**Size**	−12.21 (18.16)	0.508	-0.37 (0.045)	0.420
**Total LDSA**	−0.038 (0.014)	0.016	−7.8 × 10^−5^ (0.00003)	0.042
**LAeq8hr**	33.17 (39.56)	0.411	0.139 (0.099)	0.172
**Lc Peak**	153.03 (64.49)	0.027	0.362 (0.161)	0.035
**Age**	−92.95 (38.13)	0.024	−0.187 (0.095)	0.062
**BMI**	64.73 (92.96)	0.494	−0.40 (0.232)	0.864
**Hypertension**	−439.43 (611.93)	0.481	−1673 (1.525)	0.285
**Smoking**	−1305.9 (836.51)	0.133	−2158 (2.085)	0.312
**R^2^**	0.473		0.512	
**With Interaction Term 1 (Total LDSA × Lc Peak)**				
**Total LDSA × Lc Peak**	−0.002 (0.007)	0.739	−2.09 × 10^−5^ (1.7 × 10^−5^)	0.242
**Total LDSA**	0.283 (0.953)	0.769	0.003 (0.002)	0.255
**Lc Peak**	167.66 (78.89)	0.046	0.488 (0.190)	0.019
**Age**	−94.87 (39.37)	0.026	0.204 (0.095)	0.044
**R^2^**	0.476		0.545	

## Data Availability

The data presented in this study are available on request from the corresponding author. The data are not publicly available due to privacy policy.

## References

[1] Buonanno G., Bernabei M., Avino P., Stabile L. (2012). Occupational exposure to airborne particles and other pollutants in an aviation base. Environ. Pollut..

[2] Campagna M., Frattolillo A., Pili S., Marcias G., Angius N., Mastino C.C., Cocco P., Buonanno G. (2016). Environmental exposure to ultrafine particles inside and nearby a military airport. Atmosphere (Basel).

[3] Marcias G., Casula M., Uras M., Falqui A., Miozzi E., Sogne E., Pili S., Pilia I., Fabbri D., Meloni F. (2019). Occupational fine/ultrafine particles and noise exposure in aircraft personnel operating in airport taxiway. Environments.

[4] Boldo E., Medina S., Le Tertre A., Hurley F., Mücke H.-G., Ballester F., Aguilera I. (2006). Apheis: Health impact assessment of long-term exposure to PM2.5 in 23 European cities. Eur. J. Epidemiol..

[5] Bostan H.B., Rezaee R., Valokala M.G., Tsarouhas K., Golokhvast K., Tsatsakis A.M., Karimi G. (2016). Cardiotoxicity of nano-particles. Life Sci..

[6] (2013). IARC Monographs on the Evaluation of Carcinogenic Risks to Humans Outdoor Air Pollution.

[7] Cho W.S., Duffn R., Poland C.A., Howie S.E.M., Macnee W., Bradley M., Megson I.L., Donaldson K. (2010). Metal oxide nanoparticles induce unique infammatory footprints in the lung: Important implications for nanoparticle testing. Environ. Health Perspect..

[8] Manke A., Wang L., Rojanasakul Y. (2013). Mechanisms of nanoparticle-induced oxidative stress and toxicity. Biomed Res. Int..

[9] Pietroiusti A. (2012). Health implications of engineered nanomaterials. Nanoscale.

[10] Jia X., Hao Y., Guo X. (2012). Ultrafine carbon black disturbs heart rate variability in mice. Toxicol. Lett..

[11] Tobaldini E., Bollati V., Prado M., Fiorelli E.M., Pecis M., Bissolotti G., Albetti B., Cantone L., Favero C., Cogliati C. (2018). Acute particulate matter affects cardiovascular autonomic modulation and IFN-γ methylation in healthy volunteers. Environ. Res..

[12] Shutt R.H., Kauri L.M., Weichenthal S., Kumarathasan P., Vincent R., Thomson E.M., Liu L., Mahmud M., Cakmak S., Dales R. (2017). Exposure to air pollution near a steel plant is associated with reduced heart rate variability: A randomised crossover study. Environ. Health.

[13] Basner M., Babisch W., Davis A., Brink M., Clark C., Janssen S., Stansfeld S. (2014). Auditory and non-auditory effects of noise on health. Lancet.

[14] Héritier H., Vienneau D., Foraster M., Eze I.C., Schaffner E., Thiesse L., Rudzik F., Habermacher M., Köpfli M., Pieren R. (2017). Transportation noise exposure and cardiovascular mortality: A nationwide cohort study from Switzerland. Eur. J. Epidemiol..

[15] Lusk S.L., Gillespie B., Bonnie M.H., Ziemba R.A. (2004). Acute effects of noise on blood pressure and heart rate. Arch. Environ. Health.

[16] Kraus U., Schneider A., Breitner S., Hampel R., Rückerl R., Pitz M., Geruschkat U., Belcredi P., Radon K., Peters A. (2013). Individual daytime noise exposure during routine activities and heart rate variability in adults: A repeated measures study. Environ. Health Perspect..

[17] Sim C.S., Sung J.H., Cheon S.H., Lee J.M., Lee J.W., Lee J. (2015). The effects of different noise types on heart rate variability in men. Yonsei Med. J..

[18] Correia A.W., Peters J.L., Levy J.I., Melly S., Dominici F. (2013). Residential exposure to aircraft noise and hospital admissions for cardiovascular diseases: Multi-airport retrospective study. BMJ.

[19] Hansell A.L., Blangiardo M., Fortunato L., Floud S., de Hoogh K., Fecht D., Ghosh R.E., Laszlo H.E., Pearson C., Beale L. (2013). Aircraft noise and cardiovascular disease near Heathrow airport in London: Small area study. [Erratum appears in BMJ. 2014;348:g3504]. BMJ.

[20] Sørensen M., Andersen Z.J., Nordsborg R.B., Jensen S.S., Lillelund K.G., Beelen R., Schmidt E.B., Tjønneland A., Overvad K., Raaschou-Nielsen O. (2012). Road traffic noise and incident myocardial infarction: A prospective cohort study. PLoS ONE.

[21] Huss A., Spoerri A., Egger M., Röösli M. (2010). Aircraft noise, air pollution, and mortality from myocardial infarction. Epidemiology.

[22] Gan W.Q., Davies H.W., Koehoorn M., Brauer M. (2012). Association of long-term exposure to community noise and traffic-related air pollution with coronary heart disease mortality. Am. J. Epidemiol..

[23] Shaffer F., Ginsberg J.P. (2017). An overview of heart rate variability metrics and norms. Front. Public Health.

[24] Peters A., Hampel R., Cyrys J., Breitner S., Geruschkat U., Kraus U., Zareba W., Schneider A. (2015). Elevated particle number concentrations induce immediate changes in heart rate variability: A panel study in individuals with impaired glucose metabolism or diabetes. Part. Fibre Toxicol..

[25] Fan T., Wang Z., Su L., Fang S., Cavallari J., Byun H.M., Lin X., Baccarelli A.A., Christiani D.C. (2014). Cardiovascular responses and global DNA methylation changes after short-term occupational metal fume exposure. Am. J. Respir. Crit. Care Med..

[26] Lee M.S., Eum K.D., Rodrigues E.G., Magari S.R., Fang S.C., Modest G.A., Christiani D.C. (2016). Effects of personal exposure to ambient fine particulate matter on acute change in nocturnal heart rate variability in subjects without overt heart disease. Am. J. Cardiol..

[27] Huang F., Wang P., Pan X., Wang Y., Ren S. (2020). Effects of short-term exposure to particulate matters on heart rate variability: A systematic review and meta-analysis based on controlled animal studies. Environ. Pollut..

[28] Task Force of the European Society of Cardiology and the North American Society of Pacing and Electrophysiology (1996). Heart rate variability: Standards of measurement, physiological interpretation, and clinical use. Circulation.

[29] Weichenthal S., Kulka R., Dubeau A., Martin C., Wang D., Dales R. (2011). Traffic-related air pollution and acute changes in heart rate variability and respiratory function in urban cyclists. Environ. Health Perspect..

[30] Park S.K., O’Neill M.S., Vokonas P.S., Sparrow D., Schwartz J. (2005). Effects of air pollution on heart rate variability: The VA normative aging study. Environ. Health Perspect..

[31] Rizza V., Stabile L., Vistocco D., Russi A., Pardi S., Buonanno G. (2019). Effects of the exposure to ultrafine particles on heart rate in a healthy population. Sci. Total Environ..

[32] Schneider A., Hampel R., Ibald-Mulli A., Zareba W., Schmidt G., Schneider R., Rückerl R., Couderc J.P., Mykins B., Oberdörster G. (2010). Changes in deceleration capacity of heart rate and heart rate variability induced by ambient air pollution in individuals with coronary artery disease. Part. Fibre Toxicol..

[33] Zareba W., Couderc J.P., Oberdorster G., Chalupa D., Cox C., Huang L.S., Peters A., Utell M.J., Frampton M.W. (2009). ECG parameters and exposure to carbon ultrafine particles in young healthy subjects. Inhal. Toxicol..

[34] El Aarbaoui T., Chaix B. (2020). The short-term association between exposure to noise and heart rate variability in daily locations and mobility contexts. J. Expo. Sci. Environ. Epidemiol..

[35] Meier R., Cascio W.E., Ghio A.J., Wild P., Danuser B., Riediker M. (2014). Associations of short-term particle and noise exposures with markers of cardiovascular and respiratory health among highway maintenance workers. Environ. Health Perspect..

[36] Deyaert J., Harms T., Weeneas D., Gershuny J., Glorieux I. (2017). Attaching metabolic expenditures to standard occupational classification systems: Perspectives from time-use research. BMC Public Health.

